# Patient and clinician satisfaction and clinical outcomes of Magseed compared with wire-guided localisation for impalpable breast lesions

**DOI:** 10.1007/s12282-020-01149-1

**Published:** 2020-09-24

**Authors:** Aikaterini E. Micha, Victoria Sinnett, Kate Downey, Steve Allen, Briony Bishop, Lauren R. Hector, Elaine P. Patrick, Ruth Edmonds, Peter A. Barry, Katherine D. C. Krupa, Jennifer E. Rusby

**Affiliations:** 1 Royal Marsden NHS Foundation Trust, Royal Marsden Hospital, Downs Road, Sutton, SM2 5PT Surrey UK; 2 Institute for Cancer Research, Sutton, UK; 3 Royal Marsden NHS Foundation Trust, London, UK; 4 Bedfordshire Hospitals NHS Trust, South Wing, Kempston Rd, Bedford, MK42 9DJ UK

**Keywords:** Wide local excision, Breast cancer, Magnetic seed, Magseed, Localisation

## Abstract

**Background:**

Guide-wire localisation remains the most commonly used technique for localisation of impalpable breast lesions in the UK. One alternative is magnetic seed localisation. We aimed to investigate patient and clinician satisfaction in two consecutive cohorts, describe re-excision and positive margin rates, and explore reasons for positive margins and the implications for localisation techniques.

**Methods:**

A single-institution prospective service evaluation of two cohorts of consecutive cases of wire and then Magseed localisation was carried out. Data were collected on patient and clinician satisfaction, clinico-pathological findings, and causes of involved margins. *T* tests were used to compare continuous variables and Chi-squared test for satisfaction outcomes.

**Results:**

168 consecutive cases used wire-guided localisation (WGL) and 128 subsequent cases used Magseeds. Patients reported less anxiety between localisation and surgery in the Magseed group, and clinicians reported greater ease of use of Magseeds. There were no differences in lesion size, surgical complexity, or re-excision rate between the groups. In a subset of patients receiving standard wide local excision (i.e., excluding mammoplasties), the impact on margin involvement was investigated. There was no significant difference in radiological under-sizing or accuracy of localisation. However, specimen weight and eccentricity of the lesion were statistically significantly lower in the Magseed group. Despite this, re-excision rates were not significantly different (*p* = 0.4).

**Conclusions:**

This is the first large study of satisfaction with localisation and showed clinician preference for Magseed and a reduction in patient anxiety. It also demonstrated similar positive margin rates despite smaller specimen weights in the Magseed group. Magnetic seed localisation offers an acceptable clinical alternative to guide wire localisation. The impact on local service provision should also be considered.

## Introduction

Approximately one-third of all breast cancers are not palpable at the time of diagnosis [3] (90% of screen-detected cancers). Similarly, indeterminate and precancerous abnormalities are often impalpable, but may need surgical removal or biopsy, and localisation is required to guide this excision. A variety of localisation techniques has evolved, including guide wires, radioactive isotope and “seeds”, intraoperative ultrasound, magnetisable “seeds”, radio-frequency tags, and super-paramagnetic iron oxide particles (SPIOs), each with advantages and disadvantages [4]. In most UK units, the main method of localisation remains guide wires. Although widely used, wires bring logistical problems, requiring coordination between radiology and surgery, and often resulting in difficulties scheduling, disruption to theatre and radiology flow, and ultimately overrunning clinics and operating lists [5, 6]. Other complications with wires that have been reported include displacement and breakage [7, 8].

One of the more recent localisation techniques is the use of a metallic seed (Magseed^®^, Endomag, UK), and a technique similar to radioactive seed localisation (RSL) for primary lesion localisation. At the time of this study, the seed could be placed in the lesion up to 30 days before surgery, this has since been approved for longer term placement. Seeds have been shown not to migrate [9, 10]. They can be placed using stereotactic, tomosynthesis, or ultrasound guidance, and can be visualised well by mammography or ultrasound. Seeds are then detected intra–operatively by a handheld magnetometer (Sentimag^®^, Endomag, UK), which detects magnetic response and transforms to an audible and numerical response, to guide surgical excision. The detection zone is 30 mm, and the audible signal changes depending on the distance from the seed which provides additional guidance [5].

The Magseed appears to offer all of the advantages of RSL without the risk of additional radiation exposure and the challenges in handling and disposing. As it can be placed in the weeks or months preceding the operation, it may reduce logistical problems and the uncoupling of insertion and excision allows operating lists to run without localisation delays, thus providing flexibility of scheduling [11–14]. These benefits require further investigation to quantify. Magnetic seeds are significantly more expensive than guide wires, yet if they facilitate a more efficient workflow, eliminate delays in theatre and radiology lists, and allow for more bookings in radiology (potentially freeing up capacity to manage new patient referrals); this may prove to be cost-effective.

The aim of this study was to compare the standard practice of guide wires with Magseeds for lesion localisation in patients undergoing surgery for the removal of impalpable disease in terms of workflow [1], and to evaluate patient and clinician experience. Prior statistical calculations had suggested that 2000 patients would be required in a randomized-controlled trial to demonstrate a 5% reduction in re-excision rates. We felt that this difference was unlikely to be attained. However, for completeness and comparability with other studies, oncological outcomes are reported. In addition, we carried out an exploratory analysis of the reasons for involved margins.

## Patients and methods

After institutional approval, all patients undergoing breast-conserving surgery for impalpable lesions in a single institution (two hospital sites) between January 2018 and January 2019 were included in the evaluation.

In this two-phase cohort study, the first cohort of patients underwent localisation with the standard guide-wire and a subsequent cohort received Magseed localisation. The details of Magseed localisation and resection have been described previously [15]. A workflow study was undertaken at one of the hospital sites and this recruited 100 patients in each cohort, but for this report on satisfaction and clinical outcomes, data for both sites are presented, and hence, there are more than 100 cases in each cohort. Data were collected prospectively. Surgical excision, specimen measurements, and margin assessment were performed as standard practice. Any margin < 1 mm was considered positive. Prior to this study, a pilot phase was completed, so that the Magseed cases were not part of the radiological or surgical learning curve.

### Satisfaction

In addition to workflow, collection of patient and clinician satisfaction data was stated as an a priori endpoint in this study. At the time of planning, there was no validated questionnaire specifically designed to investigate patient or clinician satisfaction with localisation. A 5-point Likert scale, similar to that used in other studies [16] was employed. Patients were given an information sheet and paper questionnaire post-operatively, prior to discharge from hospital. They were asked to grade anxiety and comfort during and after the localisation procedure using a 5-point Likert scale. The patients were also asked to answer two questions about their preferences for scheduling of the localisation procedure. Free text comments were invited.

A 5-point Likert scale was also issued to the radiologist/radiographer and the surgeon, to be completed immediate after each localisation procedure (insertion or operation respectively) asking them to rate the ease of insertion and ease of detection.

### Clinical outcomes

This study was not powered to detect differences in rates of margin involvement or re-excision. However, for completeness and comparability, demographic data and pathology results including specimen weight, tumour size, positive margin rates, and re-excision rates were collected. We sought to explore the reasons for margin involvement and whether different localisation techniques could impact surgical outcomes and planned this a priori. Hence, data comparing radiological and pathological size, margin widths, and eccentricity of the lesion (calculated as the difference between the widest and closest radial margin) were also collected.

### Analysis methods

Clinical and patient satisfaction data and clinical results were presented using means and standard deviations or medians and ranges, according to whether the variables were normally distributed or not. Percentages and proportions were used to summarise categorical and frequency data. The unpaired *t* test was used to compare normally distributed continuous data, the Mann Whitney *U* test for non-parametric, and the Chi-squared test for frequency data. Confidence intervals are attached to those parameter estimates indicative of statistical inference for variables of interest.

## Results

### Data Summary

The two cohorts were similar in age, BMI, use of neo-adjuvant chemotherapy, and imaging modality used for localisation. More bracketed lesions were included in the wire cohort (probably because of the stipulation that lesions must be more than 2 cm apart to use more than one Magseed and get two separate signals). There were more cases of calcification-only in the Magseed cohort (Table 1).

**Table 1:** Demographic, localisation and clinical outcome data

	Wire	Magseed	*p*
**Data summary**			
*N*	168	128	
Mean age (± SD)	59.6 (± 10.9)	61.3 (± 11.6)	0.604
Mean BMI (± SD)	28.8 (± 6.6)	27.8 (± 8.4)	0.720
Neo-adjuvant treatment	21%	14%	0.087
Mean time from insertion to surgery	6 h	6 days	
**Number of Magseeds/wires**			**0.001**
1	133 (79%)	119 (93%)	
2	33 (20%)	9 (7%)	
3	2 (1%)	0 (0%)	
**Radiology guidance method**			0.834
Stereotaxis	31 (19%)	25 (20%)	
Ultrasound	137 (82%)	103 (81%)	
**Type of lesion**			**0.038**
Mass	119 (71%)	82 (64%)	
Calcifications	27 (16%)	37 (29%)	
Architectural distortion	3 (2%)	3 (2%)	
Marker coil	18 (11%)	6 (5%)	
Other	1 (1%)	0 (0%)	
Total	168 (100%)	128 (100%)	
**Accuracy of localisation**			0.553
Within 5 mm from lesion	162 (96%)	125 (98%)	
5–10 mm from lesion	4 (2%)	1 (1%)	
> 10 mm from lesion	2 (1%)	2 (2%)	
Haematoma	1	1	
**Data on size, margins, and re-excision**			
Magseed/wire retrieved with initial specimen *n* (%)	163^a^ (97%)	121^b^ (95%)	0.315
Positive radial margin (invasive or DCIS) *n* (%)	34 (20%)	31 (24%)	0.248
Re-excision	26 (16%)	22 (17%)	0.405
Median radiological size (mm)	15 (9–24)	13 (9–24)	0.220
Median pathological size (mm)	17.5 (8–30)	16 (10–26)	0.459
Median specimen weight all breast operations (g)	27 (15–49)	21 (11–36)	**0.006**
Median specimen weight WLE (g)	24 (15–41)	19 (11–32)	**0.010**

^a^five wires fell out of specimen

^b^two cases required cavity shaves to retrieve and three seeds fell out of specimen

There was no difference in complexity of breast operations between the two cohorts (*p* = 0.362). 85 and 83% of the patients had a wide local excision in the wire and Magseed cohorts, respectively. The remaining patients underwent mammoplasty with complex tissue rearrangement. There was a significant difference in the axillary operation (*p* = 0.018), reflecting the fact that more DCIS cases occurred in the Magseed cohort.

There were 11 consultant radiologists/radiographers and 7 consultant surgeons involved in the study. In 56 of 168 wire cases and 38 of 128 Magseed cases, senior trainees were the first operator, but always supervised by a consultant. All of the consultants have more than 10 years’ experience with wire guidance and had carried out 5 Magseed cases prior to enrolment in the study.

### Satisfaction: clinician and patient

**Fig. 1:**
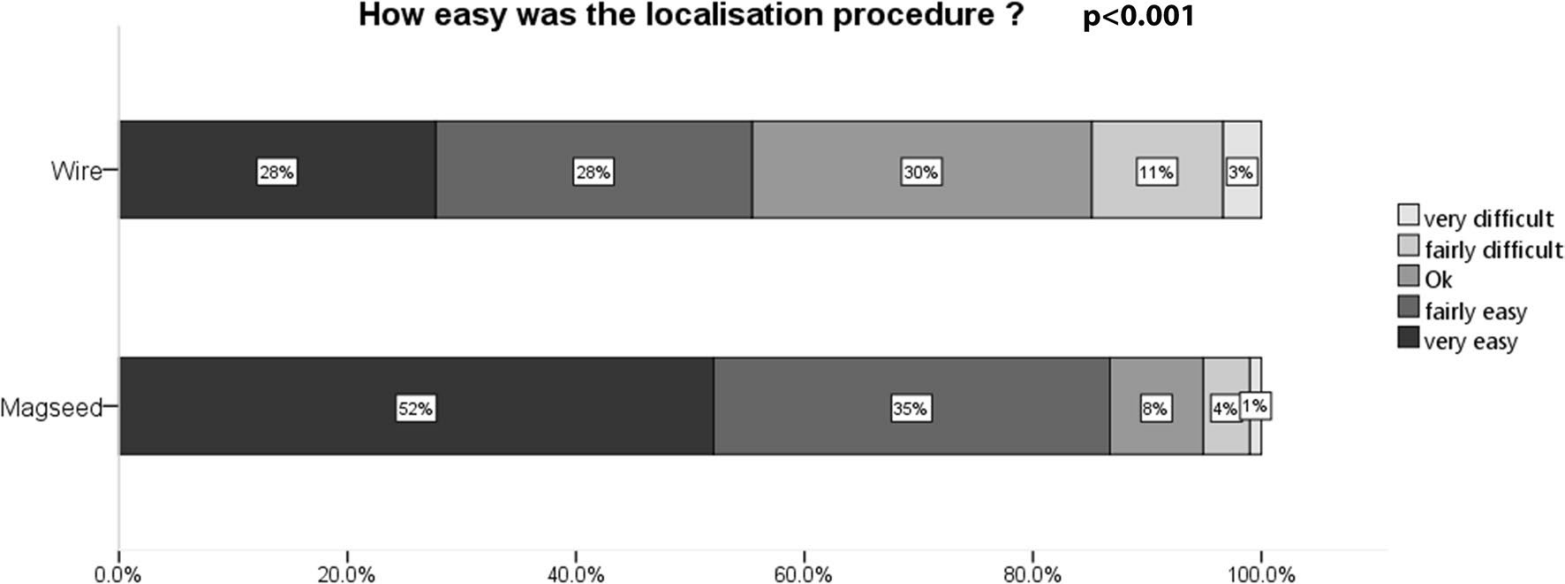
Radiologist-reported ease of radiological localisation [*N* = 148 (88%) wire cases and 98 (77%) Magseed cases]

**Fig. 2:**
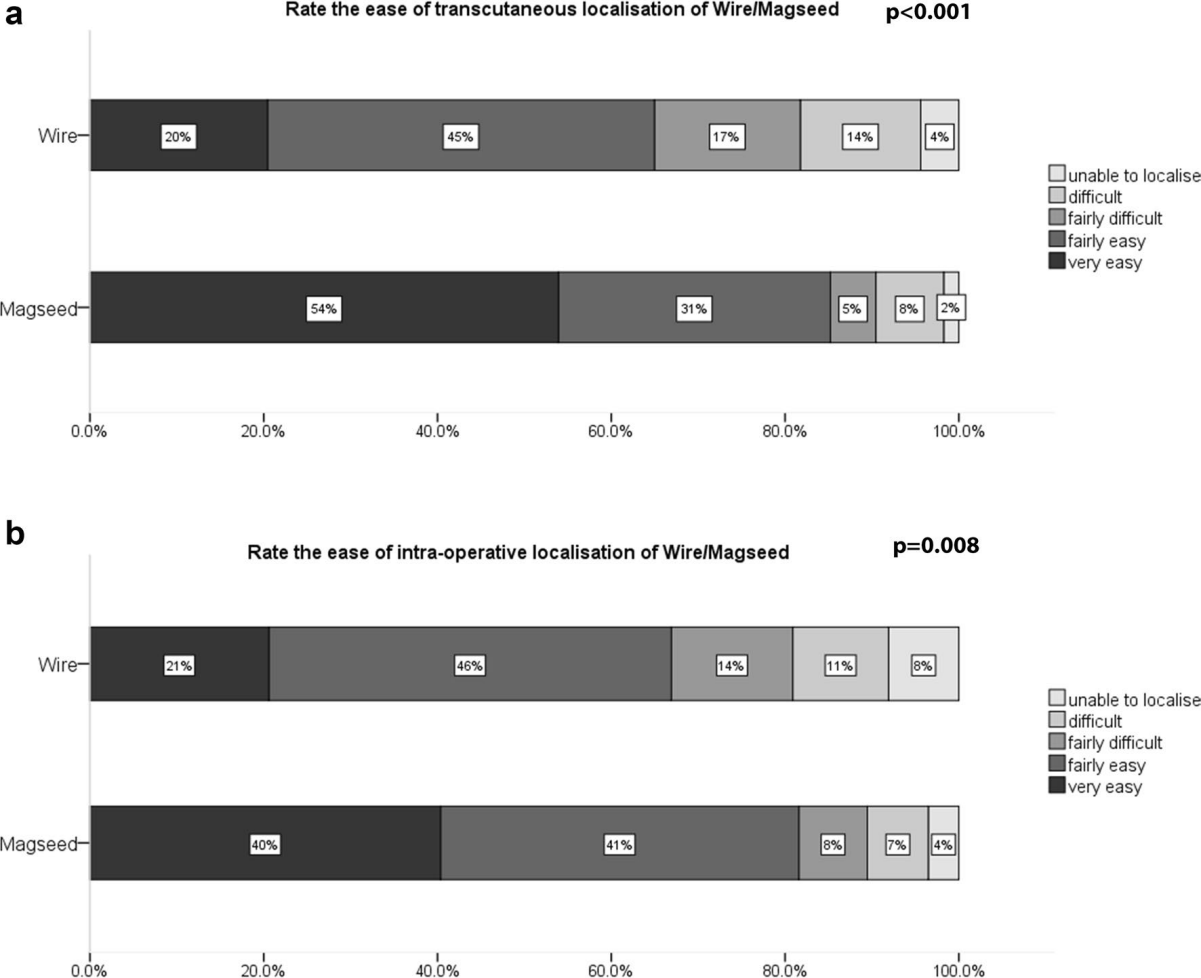
Surgeon-reported ease of transcutaneous (*N* = 137 (82%) wire cases and 115 (90%) Magseed cases) and intraoperative localisation [*N* = 136 (81%) wire cases and 114 (89%) Magseed cases]

**Fig. 3:**
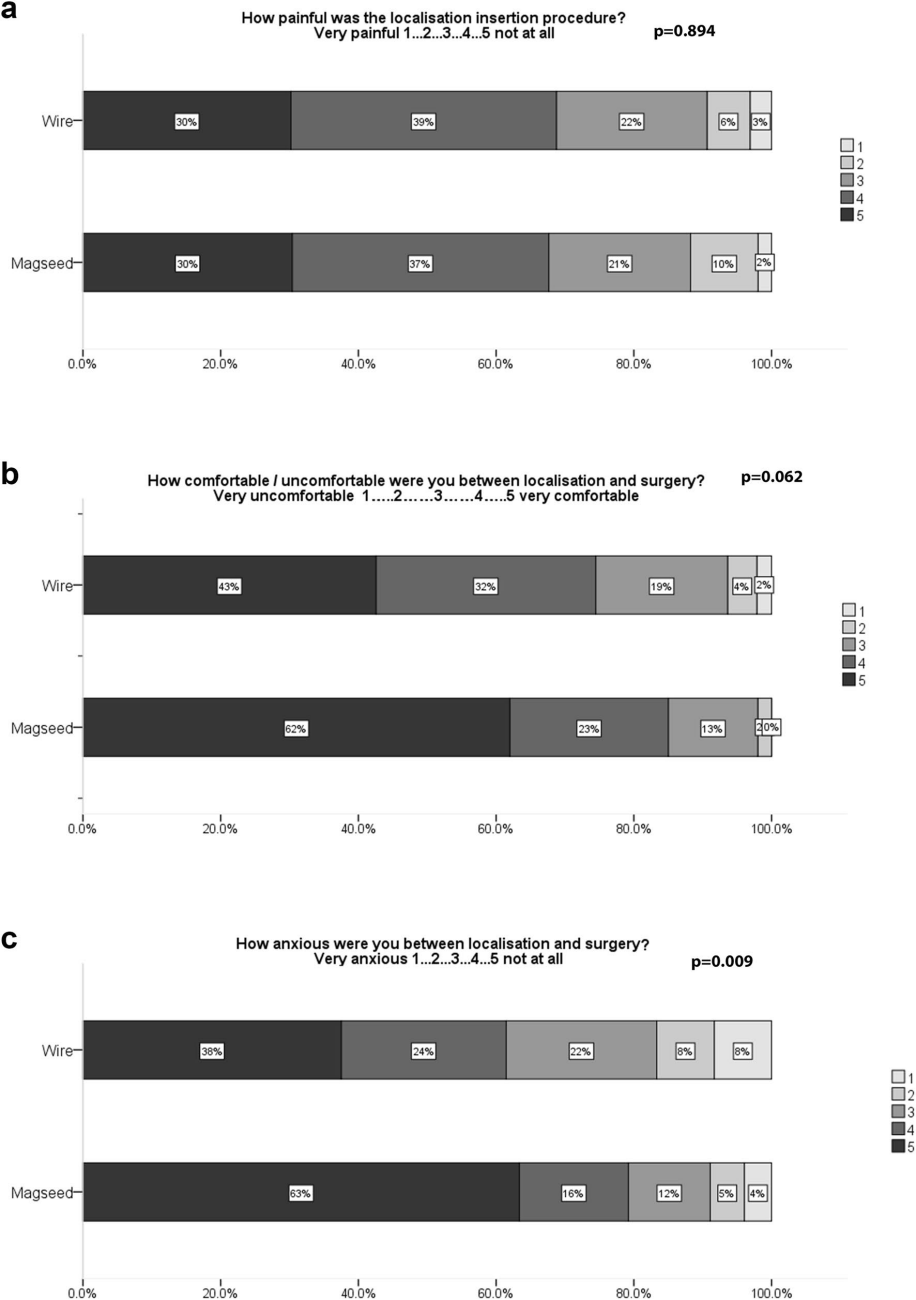
Patient-reported pain during the localisation procedure (*N* = 96 (57%) wire cases and 102 (80%) Magseed cases), comfort between localisation and surgery (*N* = 94 (56%) wire cases and 100 (78%) Magseed cases), and anxiety between localisation and surgery (*N* = 96 (57%) wire cases and 100 (78%) Magseed cases)

As with all satisfaction studies requiring completion of questionnaires, despite the intention to acquire data for consecutive cases, the data were incomplete ranging from 56 to 90% (Figs. 1, 2, 3). Nonetheless, both radiology and surgical staff were found to be statistically more satisfied with the localisation when a Magseed was used rather than a wire (Figs. 1, 2). There was a significant difference between Magseed and wire localisation patients in reported anxiety between localisation and surgery (*p* = 0.009) (Fig. 3). There was no difference in pain associated with the localisation procedure, and the difference in discomfort between that and the time of surgery between the two groups did not reach statistical significance.

**Fig. 4:**
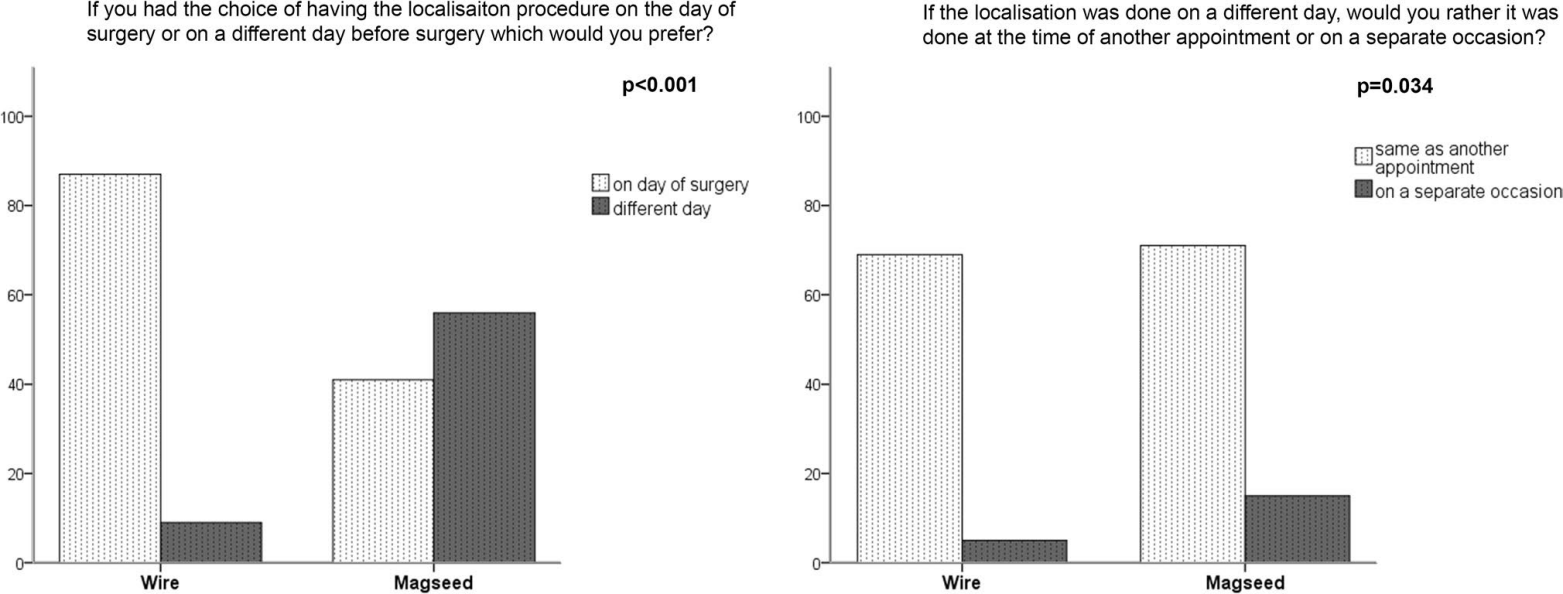
Patient preference responses to the scheduling questions. Question 1: if you had a choice of having the localisation procedure on the day of surgery or on a different day before surgery which would you prefer? *N* = 96 (57%) wire localisation cases and 97 (76%) Magseed localisation cases. Question 2: if the localisation was done on a different day, would you rather it was done at the time of another appointment or on a separate occasion? *N* = 74 (44%) wire localisation cases and 86 (67%) Magseed localisation cases

Patients who had a wire localisation were mostly satisfied with having it on the day of surgery while opinion among the women who had a Magseed was divided. The majority of both groups requested that if the insertion was done on a different day, it should be coordinated with the time of another appointment at the hospital (Fig. 4).

### Localisation results

**Fig. 5:**
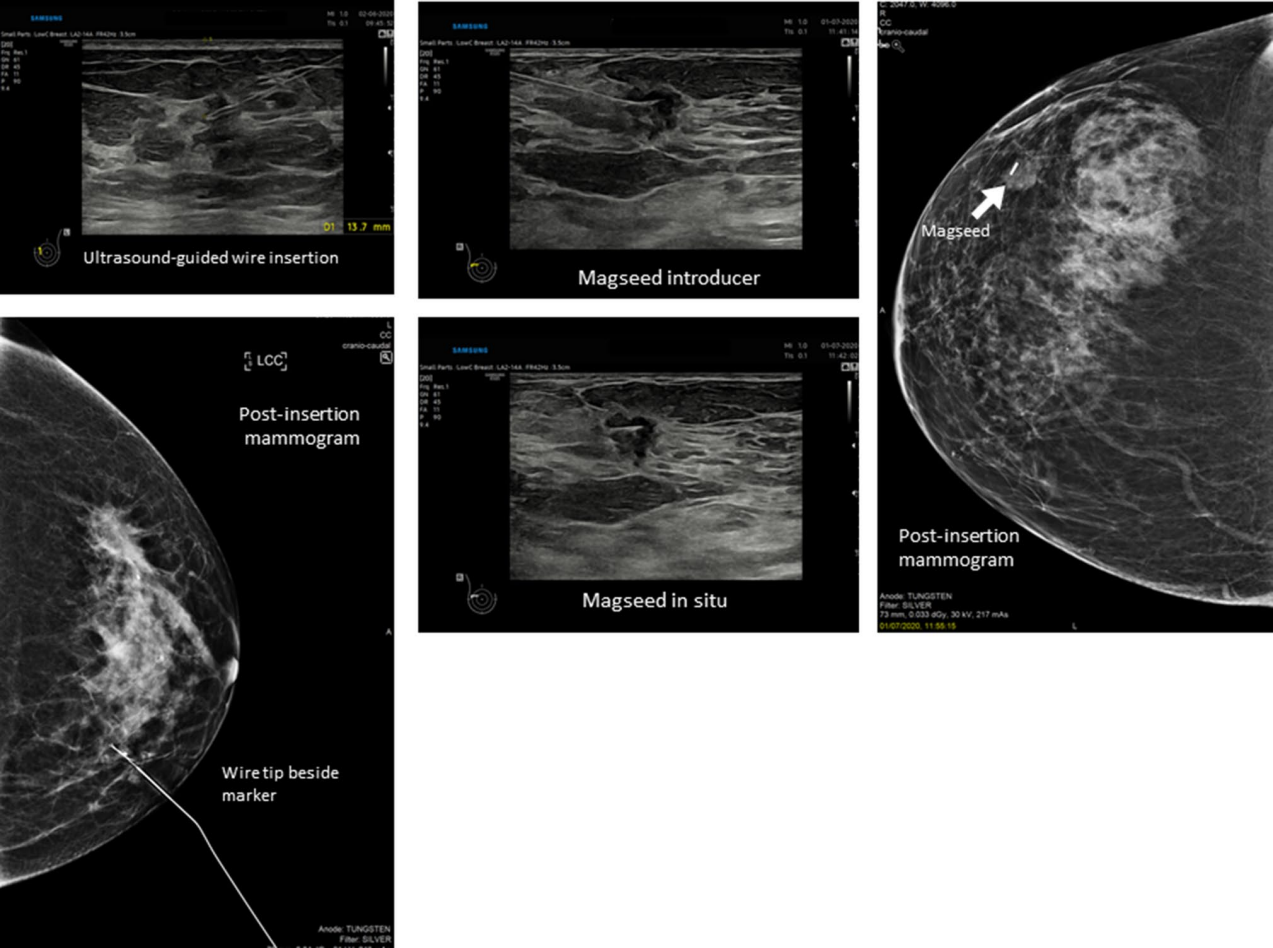
Representative images of the insertion of a wire and a Magseed

The wire and seed placement was very accurate (within 5 mm of the lesion) in 96% and 98% of the cases, respectively, which is in agreement with the previous studies. In one magnetic seed case, the marker was placed more than 10 mm from the lesion and a wire was subsequently placed to mark the correct site. Figure 5 shows representative images of the insertion of a wire and a Magseed.

Surgical excision was successful in most cases: 97% of the wires and 95% of the Magseeds were retrieved with the initial specimen. No complications were reported with the wire or the Magseed, as in the previous studies (Table 1).

### Clinical outcomes

Mean pathological and radiological size, margin positivity, and re-excision rates were similar in the two cohorts. As stated above, the study was not powered to detect a difference. Specimen weight, however, was statistically significantly lower in the Magseed group when comparing all breast operations as well as when comparing a more homogenous subgroup (wide local excisions excluding mammoplasties) (Table 1).

### Exploratory consideration of reasons for margin positivity

The reasons for margin positivity were analysed in the wide local excision subgroup, excluding those with complex oncoplastic tissue rearrangement. Factors considered were radiological under-reporting of size pre-operatively, accuracy of localisation, weight of the specimen, and eccentricity of the lesion within the specimen. As reported above, the accuracy of localisation was similar in the wire and Magseed groups and the specimen weight was statistically significantly less in the Magseed group.

#### Underestimation of size by pre-operative radiology

To evaluate the influence of pre-operative radiological underestimation of size, we calculated the difference between radiological and pathological size. No significant difference was found between wire and Magseed cohorts.

#### Eccentricity of the lesion

**Fig. 6:**
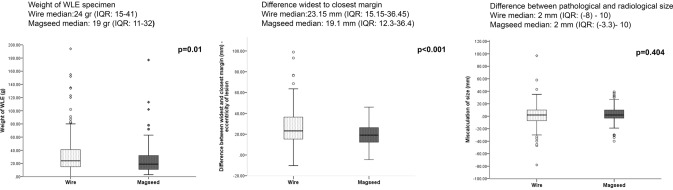
Weight of WLE, eccentricity of lesion in the specimen, and difference between pathological and radiological size

To evaluate eccentricity of the lesion in the specimen, the difference between the closest and the widest margin was calculated (mm). This difference was statistically significant (Mann–Whitney *U* test of the medians *p* < 0.001) with the Magseed-localised lesions being more central in the specimen (Fig. 6).

## Discussion

Previous studies have shown that magnetic seed localisation is feasible and safe [9, 15–19]. This is the largest series to date to report on the clinician and patient satisfaction with Magseed localisation compared with the pre-existing standard of guide wires.

### Clinician and patient satisfaction

Radiologist satisfaction was higher for Magseed localisation, and surgeons reported that both transcutaneous and intraoperative localisation was easier with Magseeds than wires. A previous small series reported high clinician satisfaction with magnetic seed localisation [16] but without comparative data.

Better patient experience has been suggested as an additional reason to consider alternative localisation techniques. To our knowledge, this is the first comparative observational study of patient satisfaction with localisation using a magnetic seed. The low proportion of patient questionnaires during the wire phase is disappointing. The study was running on two hospital sites and some patient questionnaires were missed on both sites at the start of the project (during the wire phase), but were more reliably administered as the project became routine. It is unlikely to represent any systematic bias on the part of the patients. The results for pain and comfort in the two groups are so similar that it is unlikely that a statistically significant result would have been obtained with the addition of more patient questionnaires. There was a statistically significant difference in the patient-reported Likert scales for anxiety between insertion and surgery. This is likely to be because most wire patients had localisation on the day of surgery. Eliminating the need for an additional invasive procedure, while starved, therefore, improves the patient experience in terms of anxiety. We hypothesised that patient preference responses to the scheduling questions would confirm the frequently seen phenomenon that patients are usually satisfied with the status quo, i.e., the treatment which they received (in this case, localisation on the day of surgery or on a different day). In fact, a proportion of the Magseed patients would have preferred to have all of the process completed in 1 day (localisation and surgery). The response to the second question was clearer—that women want to coordinate their appointments to reduce the number of times which they have to attend hospital, and we should be mindful that improved scheduling for clinicians should not be at the expense of patient convenience.

### Clinical outcomes

Although not powered to detect a difference, margin positivity and re-excision rates were reported for completeness. There was no significant difference between the two groups. Similar results were shown in a previous study comparing two 100-patient cohorts [20]. Margins were positive for invasive cancer or DCIS in 20.2 and 24.5% of the wire and Magseed cases, respectively; not unexpected, since the Magseed cohort contained more cases of DCIS (and not statistically significant). As a result, 15.5% of wire cases and 17.2% of Magseed cases proceeded to have re-excision to obtain clear margins. Of the patients with positive margins who did not have re-excision, all but one had only one radial margin focally involved or < 1 mm for DCIS. One patient had two radial margins involved for invasive disease, re-excision was recommended, but the patient declined. These results are comparable with the 22% who required repeat therapeutic operations after breast-conserving surgery for non-palpable lesions reported in the UK NHS Breast Screening Program [21].

### Exploratory consideration of reasons for margin positivity

We also sought to explore the reasons for positive margins and, to our knowledge, this is the first study to examine this in detail. We hypothesised that margins are found to be involved because:

1. The size of the lesion was underestimated pre-operatively,
2. The lesion was localised inaccurately,
3. Surgeons take a specimen which is too small, or
4. The lesion is not central within the specimen.

Radiological under-sizing is unaffected by the localisation technique and this was demonstrated in our study and previously [9, 17, 19, 22]. We have shown that the accuracy of placement was the same in the two cohorts (Table 1). Surgeons have the opportunity to correct for an inappropriately small specimen or eccentricity based on specimen radiography and other intraoperative techniques, hence our expectation, which was confirmed, that we would not see a dramatic difference in re-excision rates. However, the specimen weight and eccentricity of the lesion are dependent on the certainty with which the surgeon can estimate the position of the lesion intra-operatively. Both of these were statistically significantly lower in the Magseed group. Although the numerical differences are small (difference between cohorts in the mean specimen weight is only 9 g and between closest and widest margins is 8.5 mm), the outliers in the wire cohort illustrate the difficulty surgeons have with an accurate estimation of the location of the wire tip intra-operatively, leading to the unnecessary removal of healthy tissue. Evaluation of cosmetic outcome was beyond the scope of this study. However, larger resections are associated with poorer aesthetic outcome and patient satisfaction [23], so we hypothesise that changing localisation technique and thereby improving precision of surgical excision may have previously unanticipated benefits for patients.

Our study has limitations. This was a single-institution observational study with two consecutive cohorts and not a randomized-controlled trial. A randomized-controlled trial would not have permitted us to realise the potential benefits to logistics of uncoupling of localisation from surgery, and hence, consecutive cohorts were more appropriate. The two cohorts were not perfectly matched with more bracketing cases in the wire group and more DCIS without invasion in the Magseed group. The former is likely to be because of the recommendation that Magseeds should only be used for bracketing lesions that are more than 2 cm apart. The latter may simply reflect the variation in numbers of screening service referrals and a relatively underpowered study. Nonetheless, superior patient and clinician satisfaction data potentially make Magseed localisation a worthwhile investment, though further robust investigation of cost-effectiveness is required. We are not alone in believing that the learning curve is short [22] as the insertion process is the same as for many other lesion markers used in breast radiology and the Sentimag probe is used with a technique very similar to that of a gamma probe which has been the standard detection method for technetium99-guided sentinel lymph-node biopsy for more than a decade. The superior clinician satisfaction is more noteworthy given the limited prior experience with Magseeds compared with guide wires.

We asked all patients to complete the questionnaire on the day of surgery. This risks introducing recall bias for the question about the level of pain during the localisation procedure since the Magseeds were inserted a mean of 6 days earlier, while the wires were inserted 6 h pre-operatively. However, there seems no reason to expect a difference as most of the steps of the insertion are identical. Our primary interest was in whether women had been comfortable or anxious between insertion and surgery, and for that reason, the questionnaire had to be administered at the time of surgery. The fact that women felt less anxious on the day of surgery having had the localisation procedure done a few days earlier is an important finding.

The detailed analysis of potential reasons for positive margins adds a novel consideration to the literature on this subject and suggests additional, as yet uninvestigated, potential benefits of facilitation of surgery for impalpable disease.
